# Regulatory T cells can prevent memory CD8^+^ T-cell-mediated rejection following polymorphonuclear cell depletion

**DOI:** 10.1002/eji.201040671

**Published:** 2010-09-02

**Authors:** Nick D Jones, Matthew O Brook, Manuela Carvalho-Gaspar, Shiqao Luo, Kathryn J Wood

**Affiliations:** Transplantation Research Immunology Group, Nuffield Department of Surgical Sciences, University of Oxford, John Radcliffe HospitalOxford, UK

**Keywords:** Memory, Mouse, Tolerance, Transplantation, Treg

## Abstract

Accumulating evidence suggests that alloreactive memory T cells (Tm) may form a barrier to tolerance induction in large animals and humans due in part to a resistance to suppression by Treg. However, why Tm are resistant to regulation and how the Tm response to an allograft differs from that of naïve T cells, which are amenable to suppression by Treg, remains unknown. Here, we show that accelerated graft rejection mediated by CD8^+^ Tm was due to the enhanced recruitment of PMN to allografts in a mouse skin allograft model. Importantly, depletion of PMN slowed the kinetics of (but did not prevent) rejection mediated by Tm and created a window of opportunity that allowed subsequent suppression of rejection by Treg. Taken together, we conclude that CD8^+^ Tm are not intrinsically resistant to suppression by Treg but may rapidly inflict substantial graft damage before the establishment of regulatory mechanisms. These data suggest that if Tm responses can be attenuated transiently following transplantation, Treg may be able to maintain tolerance through the suppression of both memory and naïve alloreactive T-cell responses in the long term.

## Introduction

Immunological memory is one of the hallmarks of the adaptive immune response that is instrumental in the fight against infection, allowing rapid and efficient clearance of a previously encountered antigen following secondary exposure. However, in the setting of transplantation, there is now convincing evidence that memory T cells (Tm) may also contribute to the rejection of allogeneic organ transplants. For example, in clinical transplantation, alloreactive Tm have been shown to participate in both acute and chronic rejection processes in heart, kidney and liver transplant recipients [1–5]. Importantly, Heeger *et al*. have provided evidence that the presence of alloreactive Tm, pre-transplant, is linked to an increase in the incidence and severity of acute rejection episodes [6]. Tm have also been found to be resistant to leukocyte depleting agents, such as Alemtuzumab (Campath-1H), that are increasingly being used as induction therapy in transplant recipients [7, 8].

In addition to clinical evidence suggesting that alloreactive Tm contribute to allograft rejection, there are a number of reports demonstrating that alloreactive Tm may be detrimental to tolerance induction in experimental models [9–13]. For example, Adams *et al*. have shown that CD8^+^ Tm generated following virus infection can exhibit cross-reactivity to alloantigens and cause rejection of skin grafts in recipients treated with a tolerance inducing protocol [13]. We have previously shown that the perturbation in tolerance induction caused by alloreactive Tm may be explained, in part, by the finding that Tm are resistant to suppression mediated by Treg [9].

Despite a plethora of evidence supporting a role for Tm in transplant rejection and the finding that Tm form a barrier to tolerance induction, the exact differences between naïve and Tm-mediated rejection responses have yet to be elucidated. This may be explained in part through the difficulty associated with truly comparing a naïve and Tm response to an allograft, as naïve and Tm populations are likely to harbour different precursor frequencies of alloreactive T cells that may be clonally distinct, with alloreactive naïve and Tm having a different range of affinities/avidities and specificities for alloantigen, all of which may alter the observed alloresponse.

To obviate these potential problems in this study we used alloreactive CD8^+^ TCR-transgenic T cells (BM3) to characterise naïve and Tm responses to a skin allograft where the naïve and Tm existed at the same frequency and have the same affinity and specificity for alloantigen (H2K^b^). Using such a model, we demonstrate that BM3 Tm reject skin allografts more aggressively than their naïve counterparts due to the rapid recruitment of polymorphonuclear cells (PMN) to the graft. Furthermore, we show that the removal of PMN not only attenuates Tm-mediated rejection but allows donor-reactive Treg to suppress memory BM3 T cells in a manner similar to that seen during control of naïve responses.

## Results

### H2K^b^-reactive BM3 Tm mediate accelerated rejection of H2K^b+^ skin grafts

**Figure 1:**
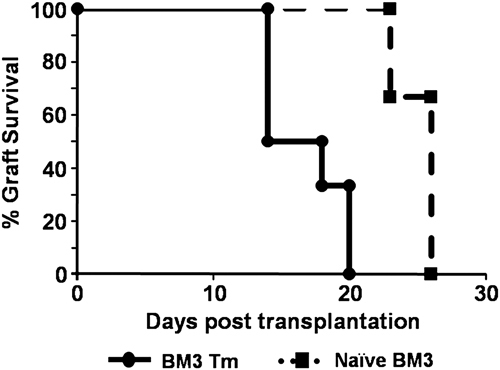
CD8^+^ BM3 Tm mediate accelerated rejection of H2K^b+^ skin grafts. 1×10^5^ naïve (square) or memory BM3 (circle) T cells were adoptively transferred to CBARAG^−/−^ mice. One day later, mice received an H2K^b+^ skin graft and the kinetics of graft rejection determined. *p*=0.01 (Log-Rank test). *n*=4 for Tm, *n*=3 for naive. Data are representative of ten independent experiments.

Alloreactive Tm can be characterised by their ability to elicit a rapid and potent recall response on re-exposure to alloantigen [10, 11, 13–15]. First, we sought to examine whether CD8^+^CD44^+^ BM3 Tm exhibited such properties and were able to reject skin allografts at a faster tempo than naïve BM3 T cells. To this end, CBARAG^−/−^ mice received 1×10^5^ naïve or memory BM3 T cells i.v. before being transplanted with an H2K^b+^ skin graft the day after cell transfer. We found that BM3 Tm were able to mediate rejection of H2K^b+^ skin grafts significantly faster than naïve BM3 T cells (Fig. 1]; median survival time=16 days and 26 days, respectively; *p*=0.01).

### Naïve and memory BM3 T cells undergo similar priming to skin allografts

**Figure 2:**
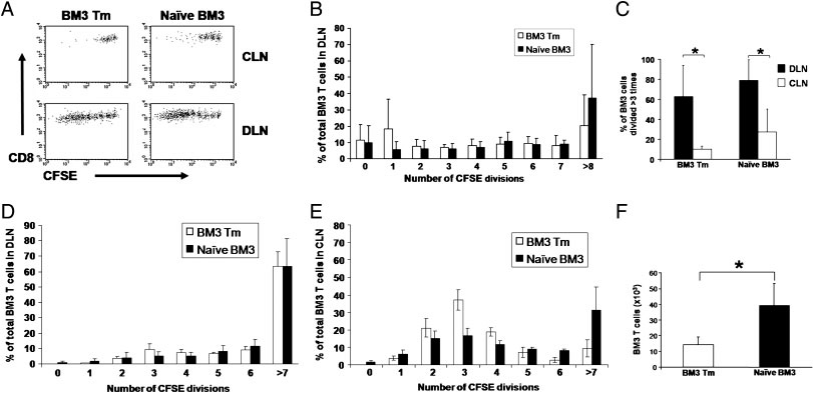
Naïve and memory BM3 T cells undergo similar proliferation in the DLN following allogeneic skin transplantation 1×10^5^ CFSE-labelled naïve or memory BM3 T cells were adoptively transferred to CBARAG^−/−^ mice. One day later, mice received an H2K^b+^ skin graft. Five (A–C) or ten (D–F) days following transplantation CD8^+^Ti98^+^TCR-β^+^ (BM3) T cells in the CLN and DLN were analysed by flow cytometry for CFSE fluorescence levels. (A) Representative CFSE *versus* CD8 dot-plots of live BM3 T cells in both CLN and DLN. Three mice/group. (B) Live BM3 T cells in the DLN of mice that had received naïve or memory T cells and a skin allograft were split according to the number of times that they had divided (judged according to CFSE dilution). Data show the mean percentage of BM3 T cells that had undergone a given division±SD by 5 days post transplantation. (C) The percentage of live BM3 T cells (±SD) that had undergone >3 divisions in the CLN and DLN by 5 days post allogeneic skin transplantation. Ten days post transplantation CFSE fluorescence levels in CD8^+^Ti98^+^TCR-β^+^ T cells in the (D) DLN and (E) CLN were used to determine the % of BM3 T cells that had divided from 1 to >7 times. (F) The absolute number of CD8^+^Ti98^+^TCR-^+^ T cells in pooled spleen, mesenteric lymph node, CLN and DLN was determined using flow cytometry. Data show mean±SD for three mice *per* group. ^*^p<0.05; unpaired Student's *t*-test.

Next, the mechanisms underlying the ability of CD8^+^ BM3 Tm to facilitate accelerated allograft rejection were examined. 1×10^5^ CFSE labelled naïve or memory BM3 T cells were transferred to CBARAG^−/−^ mice that received an H2K^b+^ skin graft 1 day later. Live BM3 T cells were analysed as shown in Supporting Information Fig. 1. By 5 days post-transplantation, both naïve and memory BM3 T cells were found to have proliferated in the DLN but not contralateral lymph nodes (CLN), mesenteric lymph nodes or spleen (Fig. 2A–C; data not shown). Further analysis revealed that both naïve and memory BM3 T cells in the DLN had undergone similar levels of proliferation as there was no difference in the rate of division between Tm and naïve BM3 T cells in response to the H2K^b+^ graft (Fig. 2B). Indeed, analysis of the percentage of cells that had passed through three or more divisions revealed a significant increase in the percentage of proliferating cells in the DLN compared to the CLN but no significant difference between Tm (62.9±31 %) and naïve (79±20.9%) BM3 T cells (Fig. 2C).

By 10 days after allogeneic skin grafting, the vast majority of BM3 T cells from mice that had received memory (63.4±9.4%) or naïve BM3 T cells (63.6±17.6%) were found to be CFSE^−^ and had therefore proliferated extensively in the DLN (Fig. 2D and E). In accordance with our previous studies, two distinct populations of T cells, one of which that had undergone few divisions as well as a population of CFSE^−^ T cells appeared in the CLN (Fig. 2E). Interestingly, recipients of naïve BM3 T cells were found to possess a higher percentage of CFSE^−^ cells in the CLN compared to recipients of BM3 Tm (31.3±13.5 *versus* 9.4±4.9%, respectively), suggesting that CFSE^−^ effector T cells derived from a Tm population had failed to undergo redistribution to the same degree as those derived from naïve cells (Fig. 2E). Furthermore, despite BM3 Tm undergoing similar levels of T-cell proliferation to that of naïve BM3 T cells, the absolute number of BM3 T cells present within the spleen, MLN, DLN and CLN of allograft recipients at 10 days after transplantation was significantly higher in mice that had received naïve (39147±13757 cells) compared to memory BM3 T cells (Fig. 2F, 14222±4972 cells, *p*<0.05).

### Similar allograft infiltration and pro-inflammatory gene expression by BM3 Tm and naïve T cells

**Figure 3:**
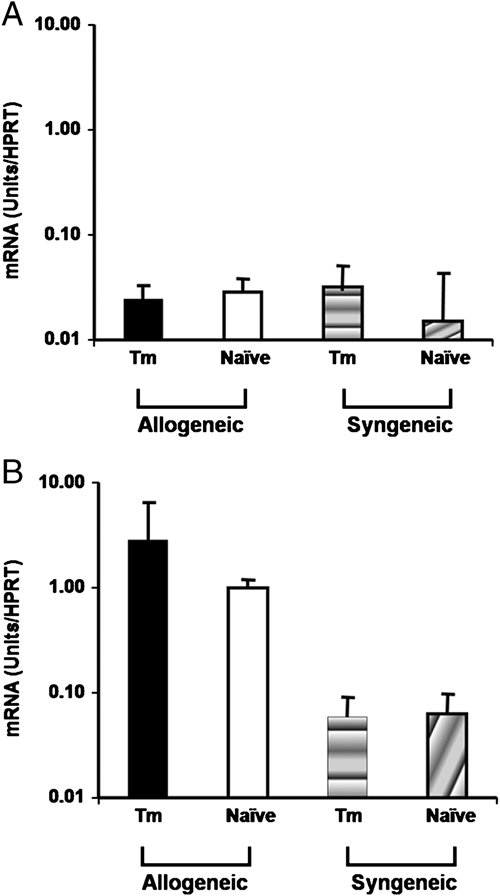
Effector T cells derived from either naïve and memory BM3 T cells infiltrate skin allografts with the same kinetics. 1×10^5^ CFSE-labelled naïve or memory BM3 T cells were adoptively transferred to CBARAG^−/−^ mice. One day later, mice received an H2K^b+^ skin graft. Five (A) or ten (B) days following transplantation skin grafts were evaluated for the expression of CD3 by quantitative RT-PCR as a surrogate marker for BM3 T-cell infiltration. Data show mean CD3 mRNA (Units/HPRT)±SD. *N*=3 skin grafts analysed *per* group *per* time-point.

**Figure 4:**
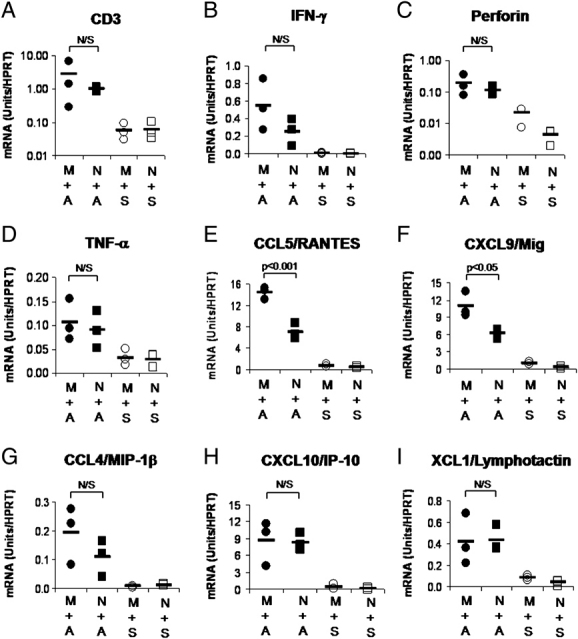
CD8^+^ T-cell infiltration and expression of effector, cytokine and chemokine genes during Tm and naïve BM3 T-cell-mediated rejection of skin allografts 1×10^5^ CFSE-labelled Tm (M) or naïve (N) BM3 T cells were adoptively transferred to CBARAG^−/−^ mice. One day later, mice received an allogeneic H2K^b+^ (M+A and N+A) or syngeneic H2^k^ (M+S and N+S) skin graft. Ten days following transplantation, skin grafts were analysed for the expression of pro-inflammatory mRNA. Data shown for mRNA expression (Units/HPRT) of (A) CD3, (B) IFN-γ, (C) Perforin, (D) TNF-α, (E) CCL5, (F) CXCL9, (G) CCL4, (H) CXCL10 and (I) XCL1. Data show three individual mice *per* group and the mean value (black dash). N/S=not significant. *P* values calculated using one-way ANOVA with Bonferroni post-test (multiple means).

We next investigated whether Tm migrated more rapidly to the allograft compared to naïve T cells, as an explanation for accelerated graft rejection by Tm (Fig. 3). Using intra-graft CD3 mRNA expression (to quantitate graft infiltrating BM3 T cells; the only T cells in these mice), we found that the level of CD3 expression in all grafts was not significantly different from that expressed in untransplanted skin (data not shown) demonstrating that no effector CD8^+^ T cells, derived from either naïve or Tm, had infiltrated grafts by 5 days post-transplantation (Fig. 3A). However, by 10 days post transplantation, BM3 T cells in both sets of mice had infiltrated the allografts, but no difference was found in the extent of infiltration determined either by CD3 expression (Fig. 3B) or by staining allografts with an anti-CD8 mAb (data not shown). Similarly, gene expression analyses revealed no difference in the intragraft expression of a number of pro-inflammatory genes such as IFN-γ, perforin, TNF-α, CCL4, CXCL10 and XCL1 between allografts taken from mice that had received either Tm or naïve BM3 T cells (Fig. 4B–D and G–I), although it was noted that mice that had received Tm were found to express a twofold increase in the levels of CCL5 and CXCL9 intragraft mRNA (Fig. 4E and F). Intragraft expression of all genes was found to be significantly increased in all allografts compared to syngeneic grafts (Fig. 4A–I).

### Tm-mediated rapid rejection is dependent on enhanced infiltration of skin allografts by PMN

**Figure 5:**
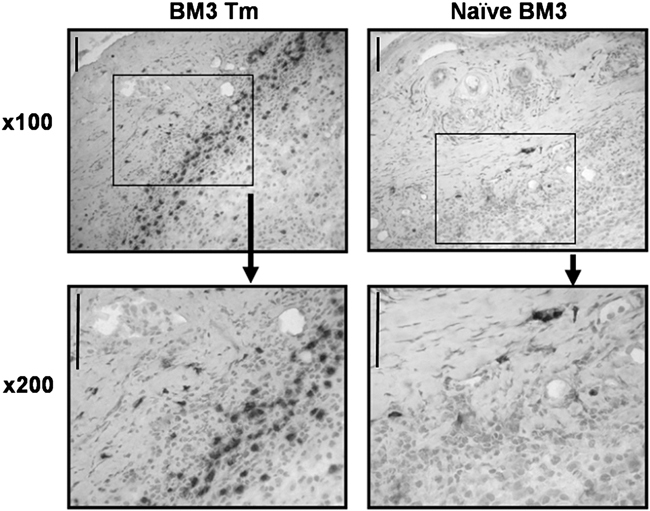
Tm-mediated rejection correlates with a diffuse infiltration of PMN by 10 days post transplantation. 1×10^5^ naïve or memory BM3 T cells were adoptively transferred to CBARAG^−/−^ mice that received an H2K^b+^ skin graft 1 day later. Ten days following transplantation skin grafts were analysed by immunohistochemistry for the presence of PMN (using an anti-Gr-1 mAb). Photomicrographs are representative of four sections *per* graft taken from three mice *per* group. Scale bars represent approximately 50 μM.

As the studies had thus far failed to reveal any significant difference between the response of naïve and Tm to an allograft, we next assessed the level of infiltration of other cell types recruited to the skin allografts during rejection to test the hypothesis that Tm may enable the recruitment of other populations of effector cells that can contribute to rejection. Skin allografts taken 10 days after transplant from mice that had received BM3 Tm were found to harbour an extensive PMN infiltrate, whereas PMN were rarely found in the grafts of mice that had received naïve BM3 T cells (Fig. 5).

**Figure 6:**
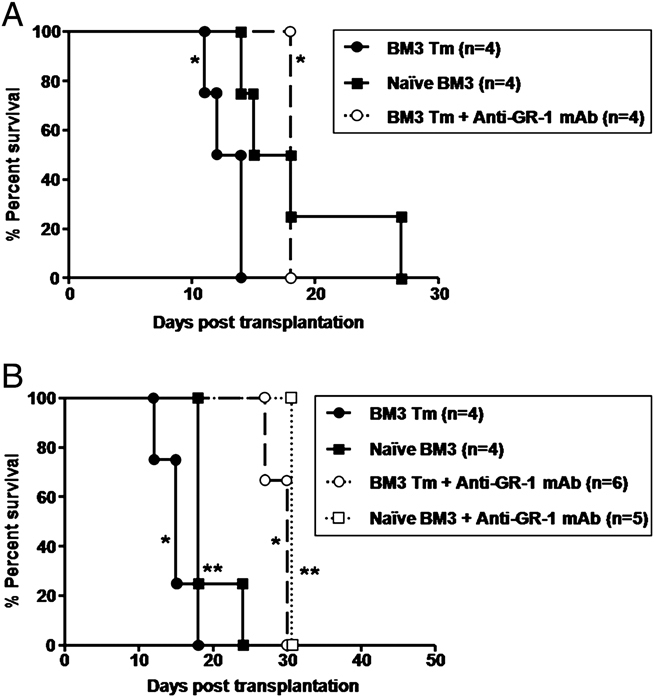
Accelerated rejection by BM3 Tm is prevented by the administration of a depleting anti-Gr1 mAb. 1×10^5^ naïve or memory BM3 T cells were adoptively transferred to CBARAG^−/−^ mice that received an H2K^b+^ skin graft 1 day later. Anti-Gr-1 mAb was administered i.p. where indicated (200 μg/dose) on days 5, 10, 15 and 20 post transplantation (A) or on days 3, 6, 9, 12, 15, 18, 21, 24 and 27 post transplantation (B). A comparison between groups labelled with either ^*^ or ^**^ denotes a significant difference between groups (*p*<0.05; Log-Rank test).

To test whether infiltrating PMN contributed to accelerated rejection, we investigated the impact of PMN depletion on allograft rejection mediated by BM3 Tm (Fig. 6). Mice that received BM3 Tm and a depleting anti-Gr-1 mAb on days 5, 10, 15 and 20 days i.p. post transplantation rejected skin allografts in a delayed fashion compared to Tm-mediated rejection without anti-Gr-1 mAb (Fig. 6A; *p*<0.05), but with the same tempo as mice that had received naïve BM3 T cells. As PMN repopulate rapidly after depletion the experiment was repeated but the anti-Gr-1 mAb was given every 3 days for 27 days. Again the accelerated rejection triggered by BM3 Tm was abrogated in mice treated with the anti-Gr-1 mAb (Fig. 6B). Additionally, allografts transplanted to mice that received naïve T cells and anti-Gr-1 mAb also enjoyed prolonged survival (*p*<0.05) with the grafts rejected with the same kinetics as those placed on mice that received BM3 Tm and anti-Gr-1 mAb.

### PMN depletion allows Treg to prevent CD8^+^ Tm-mediated graft rejection

**Figure 7:**
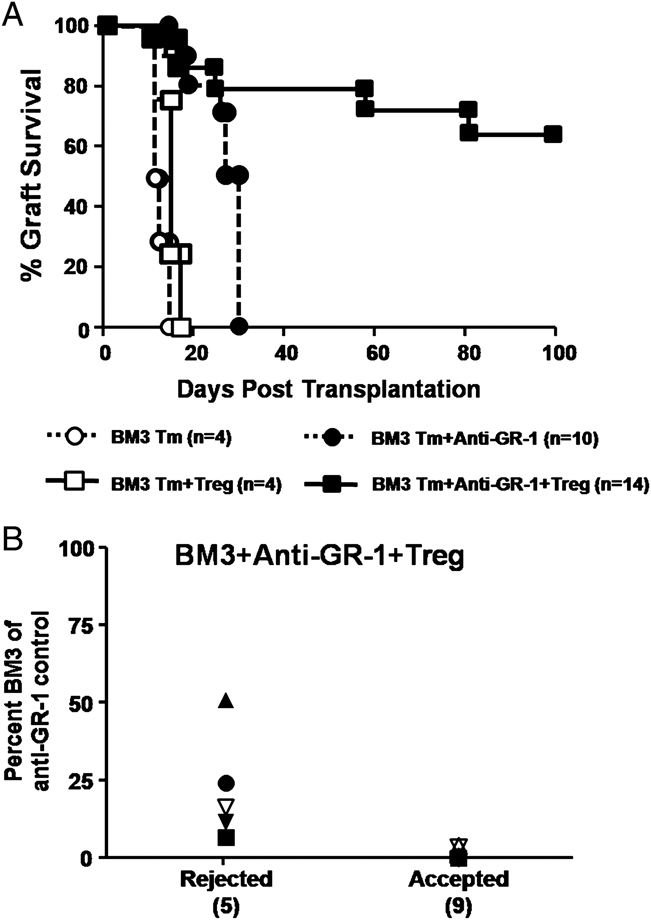
Treg suppress BM3 Tm-mediated rejection in the absence of PMN 1×10^5^ memory BM3 T cells were adoptively transferred alone or together with CD25^+^CD4^+^ T cells isolated from syngeneic mice that had received anti-CD4 mAb and donor cells to CBARAG^−/−^ mice. The following day, mice received an H2K^b+^ skin graft and anti-Gr-1 mAb was administered i.p. where indicated (200 μg/dose) on days 3, 6, 9, 12, 15, 18, 21, 24 and 27 post transplantation. (A) Skin allograft survival; data pooled from two independent experiments. (B) Number of spleen BM3 T cells at 100 days post transplantation. Data are represented as the number of BM3 T cells in mice that received anti-Gr-1 mAb and Treg as a percentage of BM3 T cells in mice that received anti-Gr-1 mAb only. Mice that received anti-Gr-1 mAb and Treg were split according to whether they had accepted skin allografts for >100 days compared to those mice where skin allografts were ultimately rejected.

We have previously shown that whilst Treg can control graft rejection mediated by naïve BM3 T cells, such cells are entirely ineffective at controlling rejection by memory BM3 T cells [9]. We hypothesised that this failure to regulate Tm responses may be due to the rapidity of rejection rather than an innate resistance of Tm to regulation. To address this question, 1×10^5^ memory BM3 T cells were transferred to CBARAG^−/−^ mice with or without 3×10^5^ CD25^+^CD4^+^ T cells that had been isolated from syngeneic mice that had received anti-CD4 and donor cells (a protocol that we have previously shown to generate donor-reactive Treg [16]). As previously demonstrated, such mice rejected skin allografts acutely despite the co-injection of Treg (Fig. 7). However, skin allografts transplanted to mice that had received BM3 Tm, Treg and additionally were depleted of PMN enjoyed prolonged survival with 65% of mice accepting the allografts long-term (median survival time>100 days; *n*=14; Fig. 7A).

Analysis of such mice 100 days post transplantation revealed that, as previously demonstrated following Treg suppression of naïve BM3 T cells [9, 17], mice that received BM3 Tm, Treg and PMN depletion had lower numbers of peripheral BM3 T cells than in mice that did not receive Treg. Mice that had received anti-Gr-1 mAb only had similar numbers of peripheral BM3 T cells to control mice (data not shown). Therefore, the extent of loss of BM3 T cells was found to correlate with graft survival, suggesting a common mechanism in the suppression of naïve and Tm responses by donor-reactive Treg.

## Discussion

Accumulating evidence suggests that Tm play an important, and previously underestimated, role in allograft rejection. Furthermore, it has been suggested that the presence of Tm before transplantation may form a barrier to the induction of tolerance [10, 13, 18, 19]. Clearly, elucidation of the mechanisms by which Tm mediate allograft rejection and resist the induction of tolerance will be key to the translation of experimental tolerance induction strategies to clinical transplantation as humans have pre-existing Tm that recognise alloantigen either as a result of heterologous immunity or sensitisation.

We have previously reported that CD8^+^ BM3 Tm mediate more rapid rejection than their naïve counterparts and are resistant to suppression by Treg [9, 14]. We therefore postulated that as an explanation for this phenomenon that Tm may show enhanced proliferation, effector function and infiltration of skin grafts after transplantation. It has previously been reported that Tm have a lower activation threshold than naïve cells resulting in the ability to respond to far lower doses of antigen [20–22] with a reduced requirement for co-stimulation [23]. Following activation, it is also clear that Tm are capable of rapidly producing effector cytokines [22, 24] and developing cytotoxicity following restimulation [25]. Finally, Tm have been shown to express differential adhesion molecules, lymphokine, chemokine and chemokine receptors compared to naive T cells [26–30]. However, a comparison of the response of naïve and memory CD8^+^ T cells to a skin allograft *in vivo*, surprisingly failed to identify any of these factors as being responsible for more rapid skin allograft rejection mediated by CD8^+^ Tm (Figs. 2–4).

In the light of these data, we propose that following allogeneic skin transplantation, Langerhans cells activated by ischemia and surgical trauma act as potent APC with full costimulatory capacity nullifying the advantage Tm normally possess when responding to antigen under conditions of suboptimal costimulation. In support of this hypothesis, when we compared the ability of naïve and memory BM3 T cells to respond to donor splenocytes *in vivo*, a situation where alloantigen is encountered in the absence of inflammatory stimuli and therefore limited costimulation, BM3 Tm proliferated and expanded more rapidly than naïve BM3 T cells (threefold greater expansion; data not shown). It should also be noted that it takes 4–5 days for skin grafts to be re-vascularised which may delay Tm entry into allografts and early expression of intragraft effector molecules, which may not be the case in models when allografts are directly vascularised [31].

Despite the similar pattern of activation, proliferation and graft infiltration of naïve and memory BM3 T cells *in vivo*, we clearly demonstrated that CD8^+^ Tm rapidly recruit Gr-1^+^ PMN into rejecting allografts, an event that immediately precedes rejection (Fig. 5). Furthermore, the depletion of Gr-1^+^ cells from the recipient was found to abrogate accelerated allograft rejection mediated by Tm (Fig. 6). Surprisingly, we found that Gr-1^+^ cells were also utilised as an effector population involved in rejection elicited by naïve CD8^+^ T cells (Fig. 6 and data not shown). Therefore, the difference in the kinetics of rejection mediated by naïve or memory CD8^+^ T cells appears to be a quantitative, kinetic difference in the utilisation of PMN as effector cells for rejection and not a qualitative difference.

The identification that PMN play a critical role in accelerated allograft rejection mediated by Tm raises the question as to how Tm are able to stimulate migration of such cells to the graft site more rapidly than naïve T cells. It has been shown that early neutrophil infiltration directly relates to the number of antigen primed CD8^+^ T cells that subsequently enter the skin in response to antigen challenge [32]. Such an interaction may occur through the upregulation of CXCR1 by CD8^+^ T cells [33]. However, in our experiments neutrophil infiltration was observed a few days prior to Tm-mediated rejection and did not correlate with increased T-cell infiltration (Fig. 4A).

It is known that neutrophils may be recruited to tissues expressing the chemokines CXCL1 and CXCL2, through expression of the chemokine receptor CXCR2 [34–37]. Indeed, CXCR2^−/−^ mice show reduced neutrophil infiltration and expression of pro-inflammatory cytokines within transplanted allografts [34]. As such, one possibility is that Tm are able to induce expression of CXCL1 or CXCL2 at the graft site. In our studies, we have found that intragraft expression of CXCL1 and CXCL2 mRNA was increased, but that there was no significant difference between naïve and Tm-mediated rejection (data not shown). It is also worth noting that BM3 Tm rapidly produce high levels of IFN-γ following reactivation and that IFN-γ can mediate neutrophil infiltration into kidneys following ischemia-reperfusion injury [38]. Therefore, we suggest that high-level peripheral secretion of IFN-γ together with the induction of intragraft CXCL1/2 expression may be required to facilitate recruitment of PMN to the graft.

Finally, we have previously shown that whilst alloreactive Treg prevent rejection by naïve BM3 T cells, they are unable to prevent BM3 Tm-mediated graft rejection [9]. We have also shown that suppression of rejection elicited by naïve T cells in this model was primarily due to the infiltration of skin allografts by Treg and the deletion of intragraft effector T cells [17]. Based on these observations, we proposed that the failure of Treg to control Tm rejection may be due to the rapidity of rejection (through the recruitment of PMN) preceding the establishment of intragraft regulation. Here, we show that attenuating rapid Tm-mediated rejection by the transient depletion of PMN was able to facilitate the induction of long-term graft survival by Treg (Fig. 7). Indeed, these data are consistent with that of El-Sawy *et al.* who showed that transient PMN depletion resulted in long-term cardiac allograft survival following costimulatory molecule blockade where endogenous alloreactive CD8^+^ Tm form a barrier to tolerance induction in this model [39]. These data therefore suggest that transient suppression of alloreactive Tm responses may enable the generation of regulatory networks that maintain tolerance even in the face of subsequent activation of alloreactive Tm. Although in these studies Tm rejection was attenuated by depletion of PMN, other approaches such as preferential Tm depletion, blockade of certain costimulatory molecules or the suppression of effector function (as shown for naïve T cells [40]) may also allow the induction of tolerance despite the presence of alloreactive Tm.

In summary, we have performed a comparative study of the mechanisms of allograft rejection utilised by BM3 CD8^+^ Tm and naïve T cells. We have demonstrated that PMN play an important role in the rapid rejection of allografts elicited by memory CD8^+^ T cells. Furthermore, depletion of PMN was found to attenuate allograft rejection mediated by BM3 Tm and allow alloreactive Treg to maintain allograft survival long-term.

## Materials and methods

### Animals

CBA.Ca RAG-1 knockout (CBARAG^−/−^) mice were a gift from Dr. D. Kioussis (Mill Hill, London, UK). H2K^b^-reactive TCR-transgenic mice (BM3; H2^k^) were provided by Professor A. L. Mellor (Institute of Molecular Medicine and Genetics, Augusta, GA, USA; [41]). BM3RAG^−/−^ were used in these studies. C57BL/6 (H2^b^) mice were originally purchased from Harlan Olac (Bicester, UK). All mice were bred and housed in the BMS-JR, Oxford, in accordance with the Animals (Scientific Procedure) Act 1986 of the UK. All experiments used mice between ages 6 and 12 wk at the time of first procedure.

### Cell isolation

#### Naïve BM3RAG^−/−^ CD8^+^ T cells

A single-cell leukocyte suspension was made from spleens and mesenteric lymph nodes harvested from BM3RAG^−/−^ TCR-transgenic mice. All CD8^+^ T cells expressed the transgenic-TCR identified using a mAb (Ti98). Cells were stained with anti-CD8-APC and anti-CD44-PE mAb (both BD Biosciences, Oxford, UK). Cells were used either unsorted (98% CD44^−^) or CD8^+^CD44^−^ BM3 T cells were purified using a FACSAria flow cytometer. Typically preparations were >99% CD8^+^CD44^−^.

#### Memory BM3RAG^−/−^ T cells

Leukocytes were prepared from CBARAG^−/−^ mice that had received 1×10^5^ BM3 T cells and C57BL/6 alloantigen (H2K^b+^; skin allograft or splenocyte injection) 50–100 days before harvest. Tm generated by either method resulted in BM3 Tm that were phenotypically and functionally identical. Cells were stained with anti-CD8-APC and anti-CD44-PE mAb. Typically, cells contained 2–3% BM3 T cells, of which>90% were found to be CD44^+^. BM3 Tm were used either unsorted or CD44^+^ BM3 T cells were purified using a FACSAria flow cytometer. Typically, cells were>95% CD8^+^CD44^+^ T cells with 1–2% contamination with CD8^+^CD44^−^ T cells.

### CFSE labelling

Single-cell suspensions were incubated for 10 min at 37°C with 10 μM CFSE (Molecular Probes), washed twice in ice-cold RPMI 1640 (Invitrogen Life Technologies), and resuspended in PBS (Oxoid) ready for i.v. injection.

### Skin transplantation

Individual full-thickness tail skin grafts were prepared to fit the graft bed on the left lateral thorax of anaesthetised recipients. The grafts were inspected regularly until they were completely destroyed, at which time the grafts were considered rejected.

### Flow cytometric analysis and mAb

Single-cell suspensions were prepared from spleen, MLN or axillary lymph nodes. Cells were incubated with Fc block (BD Biosciences) before being stained with anti-CD8-APC, anti-TCR-β-PE (both BD Biosciences) and anti-transgenic-TCR (Ti98)-biotin mAb. The Ti98-biotin mAb was developed using streptavidin-CyChrome or streptavidin-APC-Cy7 (BD Biosciences). The Ti98 hybridoma was a generous gift from Professor A. L. Mellor [42]. All samples were then acquired immediately on either a FACSort or FACSAria (BD Biosciences) and analysed using the CellQuest or DIVA software package (BD Biosciences). Analysis of the forward *versus* side scatter of a given sample enabled live cells (and beads; see below) to be clearly distinguished from dead cells and cell debris.

The depleting anti-Gr-1 mAb (RB6.8C5) was purified from a hybridoma which was a kind gift from Professor Robert Fairchild (Cleveland Clinic Foundation, Cleveland, OH, USA; [34]).

### Enumeration of cell numbers by flow cytometry

A fixed number of 6 μM synthetic fluorescent beads (CaliBRITE beads; BD Biosciences) were added to each sample. The ratio of the cell population of interest to fluorescent beads was then determined, from which the number of such cells *per* tube could be calculated. The number of cells *per* tube was then multiplied by the proportion of the sample placed into the FACS tube to result in the total number of cells *per* sample/tissue [14].

### Real-time PCR

Skin grafts were harvested, snap frozen and DNase I (Ambion)-treated. Total RNA was isolated using the Absolutely RNA Miniprep kit (Stratagene) and reversed transcribed by the Moloney murine leukaemia virus reverse transcriptase (Invitrogen Life Technologies) as previously described [43]. An additional DNase I digestion step was performed during reverse transcription to ensure samples were free of genomic contamination. Real-time quantification was performed using the ABI PRISM 7700 Sequence Detection System (PE Applied Biosystems) with either the fluorogeneic probe (hypoxanthine phosphoribosyltransferase (HPRT), CD3, IFN-γ, TNF-α and perforin) or the SYBR Green technology (HPRT, CCL4, CCL5, XCL1, CXCL9 and CXCL10). All samples were run in duplicate using the qPCR Master Mix Plus (Eurogentec) or the Brilliant SYBR Green qPCR Master Mix (Stratagene). All primers and probes for real-time PCR have been previously described [14, 43]. Samples were standardised for HPRT, and quantification of the gene of interest is given by 2^−ΔCt^, where ΔCt is obtained by calculating the difference between Ct of the gene of interest and HPRT [44].

### Immunohistochemistry

Thin frozen sections (7 μm) were cut, air-dried overnight and fixed in acetone (BDH). After inhibition/blockade of endogenous peroxidase activity and biotin, sections were incubated with anti-Gr-1-biotin mAb (BD Biosciences) and the slides developed with ABC complex (Vector Laboratories) and diaminobenzidine substrate (Sigma-Aldrich). Sections were counterstained with Gills haematoxylin (BDH) and mounted in DPX (BDH).

### Statistical analysis

Statistical analysis was performed using Student's *t*-test or one-way ANOVA with Bonferroni post-test (multiple means). Graft survival data were analysed by the log-rank test. Values for *p*<0.05 were considered statistically significant.
