# Supplementary material for: Regulatory T cells can prevent memory CD8+ T-cell-mediated rejection following polymorphonuclear cell depletion
Source: Eur J Immunol. 2010 Sep 2;40(11):3107–16. doi: 10.1002/eji.201040671 (PMC3021718; doi:10.1002/eji.201040671)
Supplement: Supplementary file 1 [file eji0040-3107-SD1.pdf]

European Journal of Immunology

Supporting Information

for

DOI 10.1002/eji.201040671

Regulatory T cells can prevent memory $CD8^+$ T-cell-mediated rejection following polymorphonuclear cell depletion

Nick D. Jones, Matthew O. Brook, Manuela Carvalho-Gaspar, Shiqao Luo and Kathryn J. Wood

© 2010 WILEY-VCH Verlag GmbH & Co. KGaA, Weinheim

Supporting Information

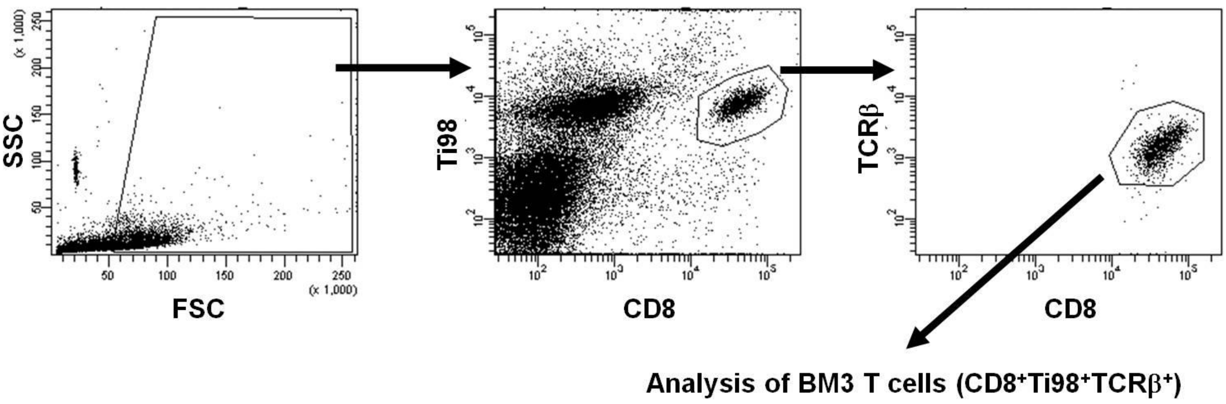

Gating strategy used to identify BM3 T cells (in relation to Figure 1 and 7B).
A single cell suspension of leuokocytes was prepared and stained with anti-CD8-APC, anti-clonotypic-TCR-Biotin (Ti98; Streptavidin-APC-Cy7 used as second stage) and TCRβ-PE. A gate was placed around live leukocytes according to the SSC/FSC profile. BM3 T cells were subsequently identified by gating on the $Ti98^{+}CD8^{+}$ population before gating on the $TCR\beta^{+}CD8^{+}$ cells within this population. These cells were analysed as BM3 T cells.
